# Preliminary results in anterior cervical discectomy and fusion with the uncovertebral joint fusion cage in a goat model

**DOI:** 10.1186/s12891-021-04412-4

**Published:** 2021-07-17

**Authors:** Yi-Wei Shen, Yi Yang, Hao Liu, Ting-Kui Wu, Li-Tai Ma, Lin Chen, Ling-Yun Hu, Chen Ding, Xin Rong, Bei-Yu Wang, Yang Meng, Ying Hong

**Affiliations:** 1 Department of Orthopedic surgery, West China Hospital, Sichuan University, No. 37 Guo Xue Rd, Chengdu, 610041 China; 2 Department of Operation Room and Anesthesia Center, West China Hospital, Sichuan University, No. 37 Guo Xue Rd, Chengdu, 610041 China

**Keywords:** Luschka joints, Uncovertebral joint fusion, Cages, Cervical spine, Goats, ACDF

## Abstract

**Objective:**

To preliminarily evaluate the safety and efficacy of the uncovertebral joint fusion cage in a goat model of cervical spine interbody fusion.

**Methods:**

Twenty-four healthy adult goats were randomly assigned to one of the two following groups: Group A, goats were implanted with an uncovertebral joint fusion cage combined with a local autograft and Group B, goats were implanted with a non-profile cage filled with a local autograft. The goats were prospectively evaluated for 24 weeks and then were sacrificed for evaluation. X-rays, CT and micro-CT scanning, and undecalcified bone histological analysis were used for the evaluation of fusion.

**Results:**

75.0% (9/12) of the goats in Group A were evaluated as having fusion at 12 weeks, compared to 41.7% (5/12) in Group B. 83.3% (10/12) of the goats in Group A were evaluated as having fusion at 24 weeks compared to 58.3% (7/12) in Group B. The fusion grading scores in Group A were significantly higher than that in Group B both at 12 weeks and 24 weeks (*P* < 0.05). Micro-CT scanning and undecalcified bone histological analysis showed that new bone formation can be obviously found in the bilateral uncovertebral joint. The bone volume fraction (BV/ TV) in Group A (23.59 ± 4.43%) was significantly higher than Group B (16.16 ± 4.21%), with *P* < 0.05.

**Conclusions:**

Preliminary results of this study demonstrated that uncovertebral joint fusion cage is effective for achieving early bone formation and fusion without increase of serious complications.

## Introduction

Anterior cervical discectomy and fusion (ACDF) is the most common surgical procedure in the treatment of spondylotic radiculopathy and myelopathy with demonstrated long-term clinical success [1, 2]. Since its introduction in 1958 by Cloward [3], Robinson and Smith [4], a variety of attempts has been conducted for the refinement of the operation to minimize complications and improve interbody fusion rate. Solid fusion is of critical importance in the achievement of expected outcome in ACDF. In an effort to improve on fusion rates after ACDF, an extensive body of literature has emerged investigating various of cages and bone graft [5–7]. Despite the advancements in type and material of cage and bone graft, the method of bone grafting in the central cavity of the cage remained unchanged.

The uncovertebral joint, or the Luschka joint, is an anatomical structure unique to the cervical vertebrae from C3 to C7, sometimes to T1 or T2, which consists of uncinate process in the posterior craniolateral edges of the vertebral body, the corresponding beveled surfaces on the inferior aspect of the vertebrae above and the surrounding soft tissue connections [8]. The uncovertebral joint has been reported to play an essential role in the mobility and stability of spinal motion segments, especially lateral bending [9, 10]. In our clinical practice, significant bony fusion is often observed in the uncovertebral joint area during anterior intervertebral release procedure in patients with old cervical fracture and dislocation. Furthermore, in a long-term study for cervical artificial disc replacement, high-grade paravertebral ossification was predominantly distributed at bilateral uncovertebral joint [11]. Previous clinical research concerning the application of a Zero-profile anchored spacer (Zero-P, Johnson & Johnson) in ACDF showed that bone grafting in the uncovertebral joint region can increase fusion rate [12]. Therefore, we postulated that uncovertebral joint fusion may have potential advantages in cervical spine interbody fusion and designed a novel uncovertebral joint fusion cage. This study was performed to preliminarily evaluate the safety and efficacy of the uncovertebral joint fusion cage in a goat model for the cervical spine interbody fusion.

## Materials and methods

### Study design

The animal study was approved by the ethics committee of West China Hospital, Sichuan University, China, before beginning the experiments. Twenty-four healthy adult goats with weight ranging from 35 kg to 45 kg were included in the study. The goats were randomly assigned to one of the following groups using the software SPSS 22.0 (SPSS Inc., Chicago, IL, USA): Group A (*N* = 12), the uncovertebral joint fusion cage filled with a local autograft; Group B (*N* = 12), the non-profile cage (Shandong Kangsheng medical Devices Co., Ltd., Tai’an, China) filled with a local autograft. All the goats were prospectively evaluated for 24 weeks and then sacrificed for a micro-CT and histologic analysis.

### Implants

**Fig. 1:**
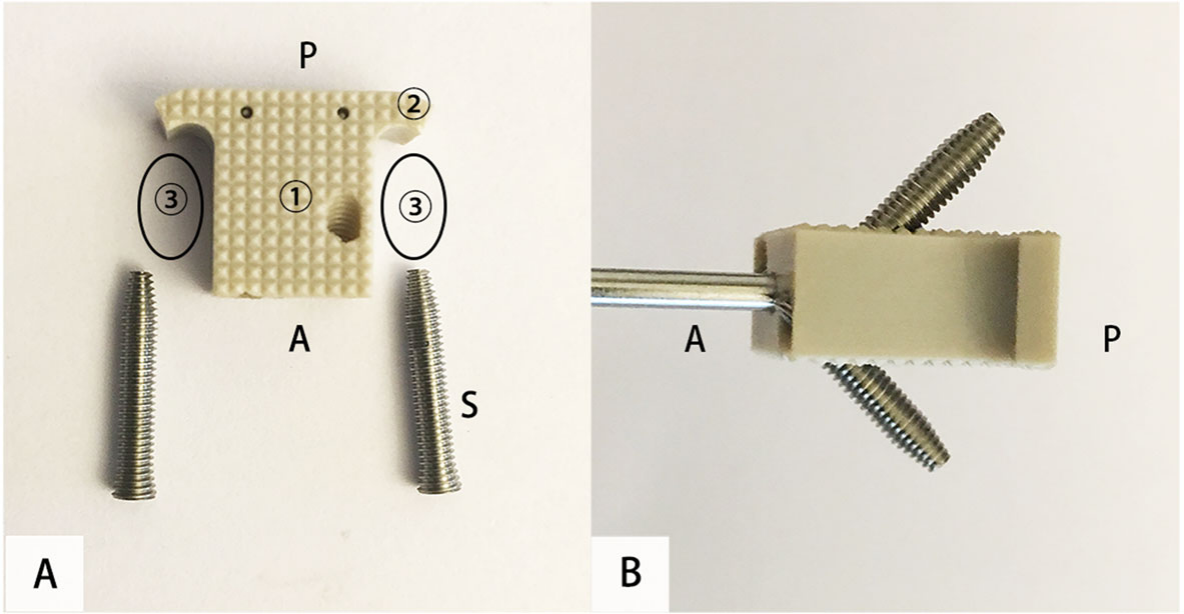
Images of the uncovertebral joint fusion cage before (**A**) and after screws fixation (**B**). 1) the polyetheretherketone (PEEK) body; 2) the two wings of this cage; 3) the bone-grafted region in the region of the bilateral uncovertebral joint; A, anterior; P, posterior

**Fig. 2:**
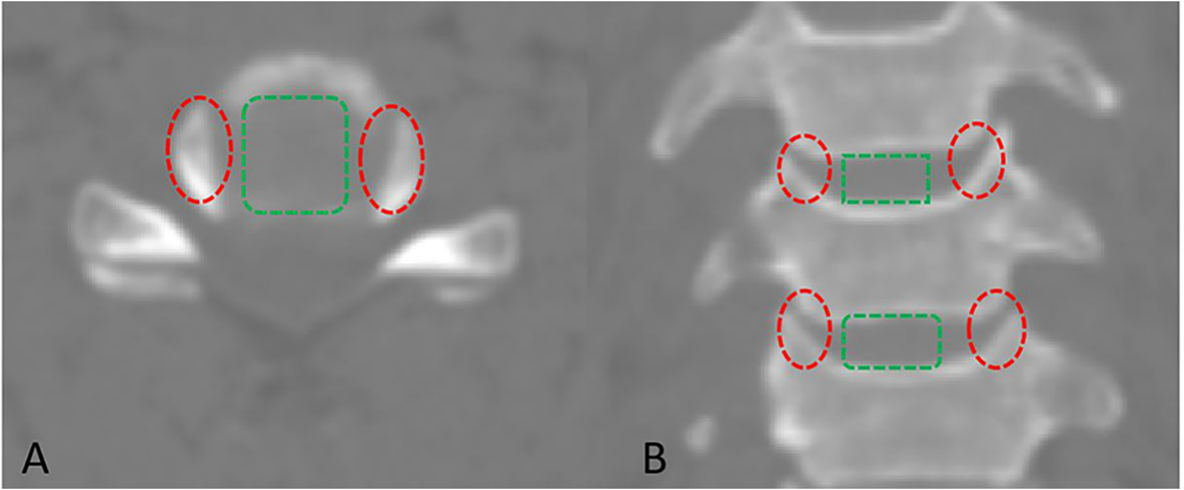
Bone graft region of the traditional cage (square box) and the uncovertebral joint fusion cage (oval box)

**Fig. 3:**
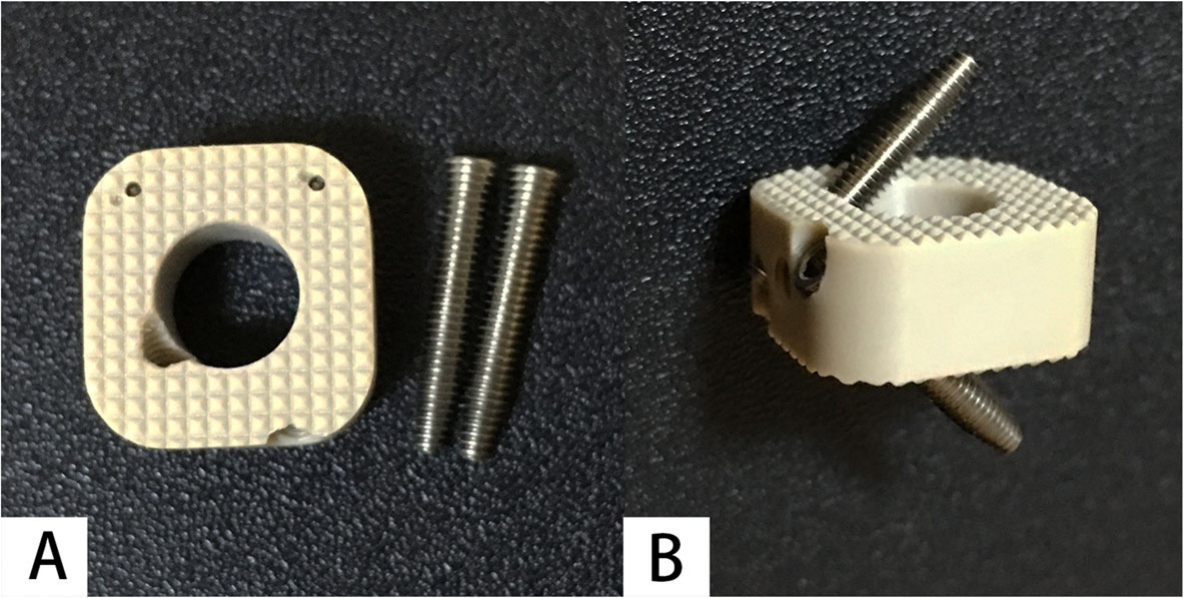
The non-profile cage used in the control group. The cage consists of a PEEK spacer with two titanium alloy screws for fixation

The uncovertebral joint fusion cage consists of a polyetheretherkeone (PEEK) spacer with two wings and two titanium alloy screws (Fig. 1). The wings are designed to prevent the graft from slipping backwards into the spinal canal and the bone graft cavity is in the regions of bilateral uncovertebral joint (Fig. 2). The bone graft cavity of non-profile cage in the control group remains in the central region as traditional cages, consisting of a PEEK spacer with two titanium alloy screws for fixation (Fig. 3).

### Surgical technique and postoperative care

**Fig. 4:**
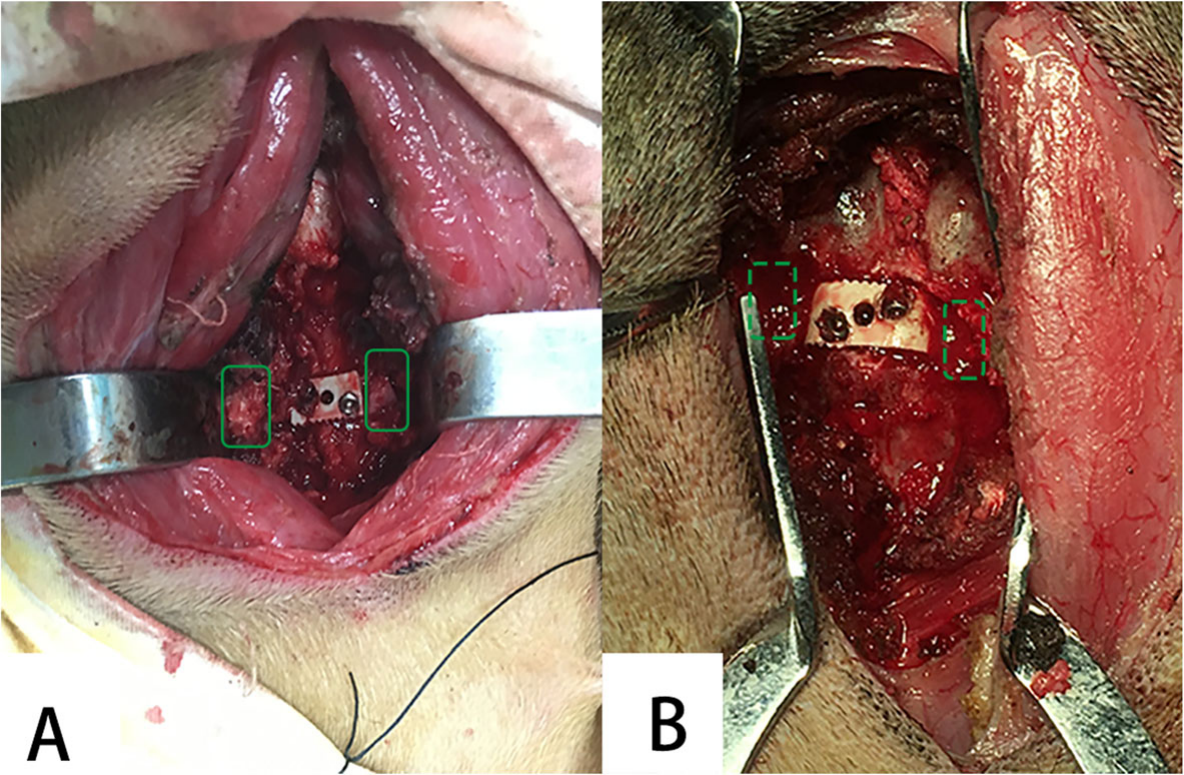
Anterior intraoperative view. The uncovertebral joint fusion cage (**A**) and the non-profile cage (**B**) were implanted at the C3–4 level using standard ACDF techniques: a local autograft (solid line box) was grafted in the uncovertebral joint region in Group A and no grafting (dashed box) was found in the uncovertebral joint region in Group B

All solid foods were avoided for a minimum of 24 h but water was allowed before the operation. In addition, 1000 mg of cefazolin sodium was injected intravenously for perioperative prophylaxis. Each animal was positioned supine with the head and neck hyperextended after induction of general anesthesia. A longitudinal skin incision was carried out to reach the cervical spine through a standard right anterolateral approach. The longus colli was elevated bilaterally using an eletrotome for exposure at the C3–4 level. After confirmation of the segment, the C3–4 discectomy was performed, and then distraction was achieved with a Caspar distractor. A spinal curette combined with a high-speed drill was used for the preparation of the cartilaginous endplate of each vertebral body. The posterior longitudinal ligament was resected with a rongeurs and the decompression was made to reach bilateral uncovertebral joints. In Group A, an uncovertebral joint fusion cage of an appropriate size was implanted into the intervertebral space. A local autograft was grafted in the region of the bilateral uncovertebral joint, and two titanium screws were placed into C3 and C4 vertebrae respectively for initial stabilization of the cage; In Group B, an appropriate non-profile cage filled with a local autograft was implanted into the C3–4 disc space without bone grafting in the uncovertebral joint region, and two titanium screws were used for the initial stabilization of the cage (Fig. 4). The wound was irrigated and closed in a layer-by-layer fashion without drainage tube insertion. The goats were transferred to a metabolic cage for observation after extubation for the first week and then transferred to open pastures until the study was completed. Each goat received 1000 mg of cefazolin sodium intravenously per day for the first week. The goats were observed for 24 weeks and then sacrificed for a Micro-CT and histologic analysis after X-rays and CT scan examinations.

### X-rays and CT scans

Lateral and posterior-anterior cervical X-rays were performed at 10 days, 12 weeks and 24 weeks after surgery when goats were awake and fixed in a neutral position. The X-rays were used for assessment of gross changes in cages and screw position, screw loosening or pullout and other complications. The cervical CT scans with sagittal and coronal reconstructions were also performed at 12 and 24 weeks after surgery with the goats receiving appropriate dose of disoprofol (10 ml to 20 ml per goat). The presence of bony trabeculation at the C3–4 level, namely bridging bone formation across the superior and inferior vertebra in the central place of the cage, outside the cage or in the uncovertebral joint region, was all defined as successful fusion in the CT scan [13, 14]. In addition, the CT scan fusion grading system (grade ranging from 0 to 3) proposed by Goldschlager et al. [15] was used for evaluation of the fusion quality (Table 1).

**Table 1:** A CT scoring system of the interbody fusion proposed by Goldschlager et al.

Fusion grade	Description
Grade 0	No new bone formation
Grade 1	New bone formation but not continuous between C3 and C4 (cleft of discontinuity)
Grade 2	Continuous bridging new bone but comprises < 30% of fusion area
Grade 3	Continuous bridging new bone formation of > 30% of fusion area

### Micro-CT scans

When the X-rays and CT scans were performed at 24 weeks after operation, all goats were sacrificed for further micro-CT scan and histologic analysis. The complete vertebrae of (C3-C4) were excised and the surrounding muscles and ligaments were removed. After fixation in 10% neutral buffered formalin at least for 1 week, all specimens were further trimmed. The superior half of the C3 vertebrae and the inferior half of the C4 vertebrae were removed. The trimmed specimens were then scanned using the Quantum GX micro-CT imaging system (PerkinElmer, Inc. Waltham, United States). The scan was performed at 90 kV, 500 mA and a spatial resolution of 30 mm. The 3D analysis software Amira 6.0.1 for Windows (Thermo Scientific™) was used for 3D images reconstruction and quantitative analysis. The region of the C3–4 intervertebral space containing the cages was chosen as volume of interest (VOI). The bone volume fraction (BV/TV) was calculated as bone volume/(total fusion mass volume - total cage volume). The screw volume was excluded in the calculation of the total volumes.

### Histological analysis

An undecalcified histological evaluation was performed for the trimmed C3–4 segments after micro-CT scanning. The specimens were dehydrated with increasing concentrations of ethanol after fixation in 10% neutral buffered formalin at least for 1 week. The specimens were then embedded without being decalcified in methylmethacrylate. Longitudinal sections in the coronal plane were prepared and stained with Stevenels blue and Van Gieson’s picro fuchsin staining used for analysis. The software Image Pro Plus 6.0 (IPP 6.0, Media Cybernetics, Inc.) was used for all quantitative analysis. The trabecular bone area fraction was calculated as the trabecular bone area/ (total fusion mass area – total cage area). The screw area was excluded when calculating the total area in analysis.

### Statistical analysis

SPSS 22.0 (SPSS 22.0, IBM Analytics) was used for all statistical analysis. The fusion scores from CT scans were compared between the two groups using a Mann-Whitney or Fisher’s Exact test. The BV/ TV and trabecular bone area fraction were compared between the two groups using an independent-samples t-Test. Statistically significant differences were defined at a 95% confidence level in this study.

## Results

### Surgical procedure

Twelve uncovertebral joint fusion cages were implanted in Group A, and 12 non-profile cages were implanted in Group B as the control group. One goat in Group B suffered from spinal cord injury along with massive blood loss during operation and died at the third day after operation. This animal was excluded, and another goat was recruited to Group B of the study. No intraoperative or postoperative complications such as wound infections, neurologic deficits or massive blood loss were observed in the remaining goats.

### X-rays and CT scan

**Fig. 5:**
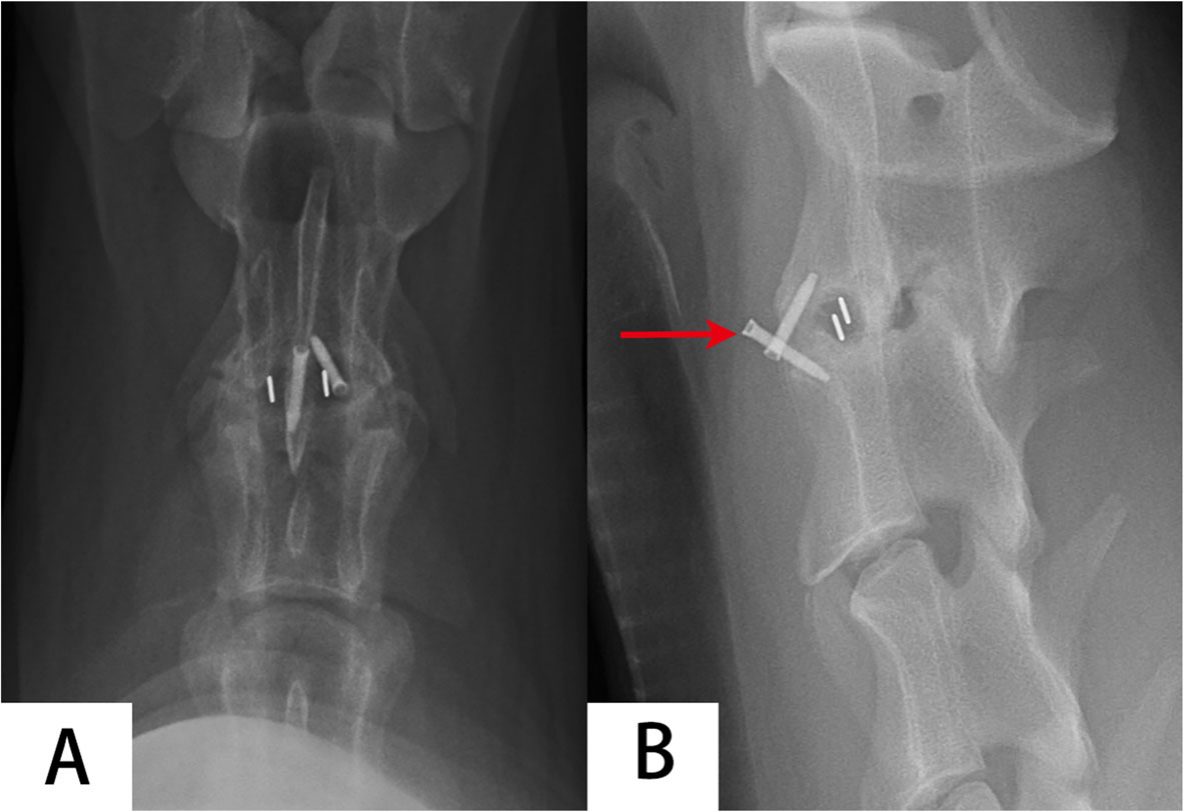
Screw pullout was observed in the non-profile cage group at 24 weeks after operation. **A** anteroposterior; **B** lateral

**Fig. 6:**
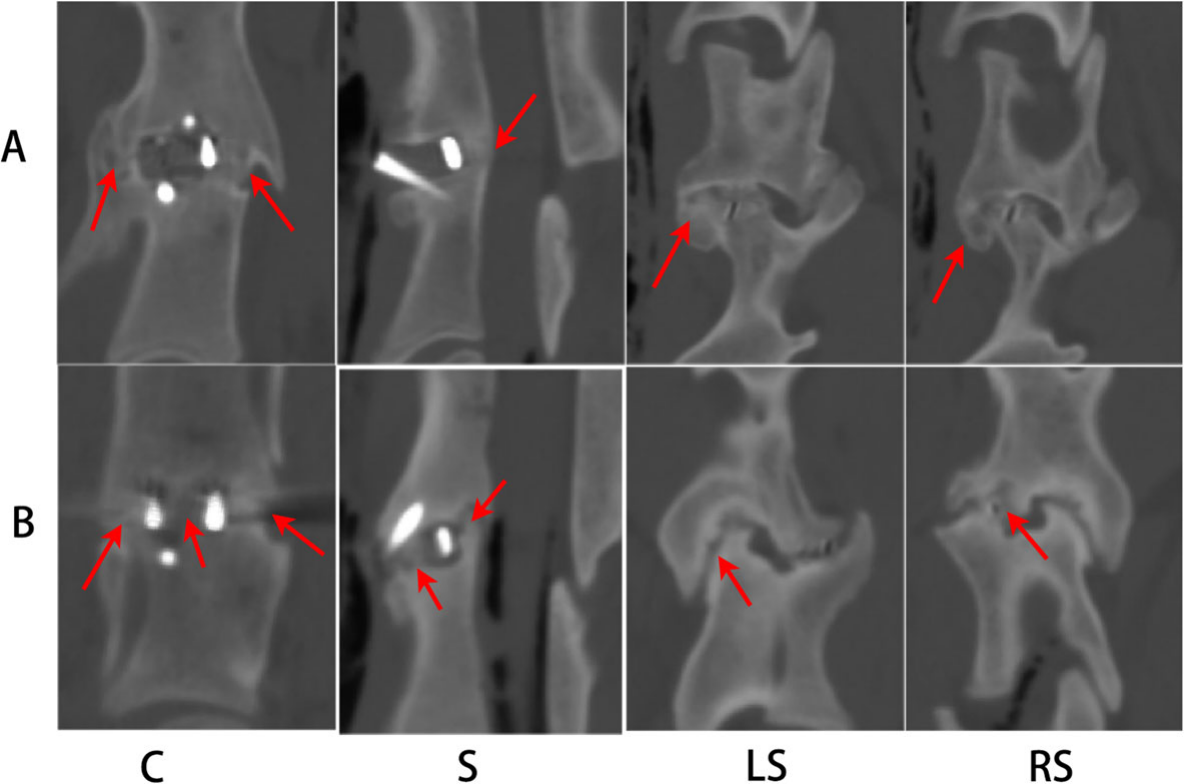
3D reconstruction images at 24 weeks after operation in the coronal and sagittal planes of the uncovertebral joint fusion cage (**A**) and the non-profile cage (**B**). A large amount of bone formation and definite fusion were detected in bilateral uncovertebral joint (arrows in C, RS and LS * A) and in back of the uncovertebral joint fusion cage (arrows in S* A); bone formation was detected inside and outside the non-profile cages (arrows in C, S, RS and LS * B). C, coronal plane; S, sagittal plane; LS, left side sagittal plane; RS, right side sagittal plane

Screw pullout (Fig. 5) was observed in one goat in Group B at 3 months after operation which did not have impact on the goat’s ability to drink and feed. Complications of cage subsidence, cage displacement, cage breakage, screws breakage were not observed in any goats. All goats successfully recovered after CT and no goat died during CT scanning. 3D reconstruction images in the coronal and sagittal planes showed obvious bone formation in the region of the bilateral uncovertebral joint in Group A (Fig. 6A), and different amounts of bone formation inside and outside of the cages in Group B (Fig. 6B).

At 12 weeks after operation, 75.0% (9/12) of the goats in Group A were evaluated as fusion (fusion score ≥ 2, Grade 2 or 3) according to the fusion grading system described previously, compared to 41.7% (5/12) in Group B. One goat in Group A, and three goats in Group B were evaluated as Grade 0 due to the absence of new bone formation in C3–4. Two goats in Group A, and four goats in Group B were evaluated as Grade 1 because no continuous new formation between C3 and C4 was detected. The CT scan fusion grading scores at 12 weeks after surgery (Table 2) show significant differences between the two groups (*P* = 0.039). At 24 weeks after operation, 83.3% (10/12) of goats in Group A were evaluated as fusion (fusion score ≥ 2, Grade 2 or 3), compared to 58.3% (7/12) in Group B, according to the fusion grading system. The CT scan fusion scores at 24 weeks after surgery (Table 3) indicate a significant difference between the two group (*P* = 0.045).

**Table 2:** Fusion grading scores in two groups at 12 weeks after surgery

Fusion score	Group A(*N* = 12)	Group B (*N* = 12)
0	1	3
1	2	4
2	3	4
3	6	1

*P* = 0.039, Mann-Whitney test

**Table 3:** Fusion grading scores in two groups at 24 weeks after surgery

Fusion score	Group A(*N* = 12)	Group B (*N* = 12)
0	0	2
1	2	3
2	3	5
3	7	2

*P* = 0.045, Mann-Whitney test

### Micro-CT scans

**Fig. 7:**
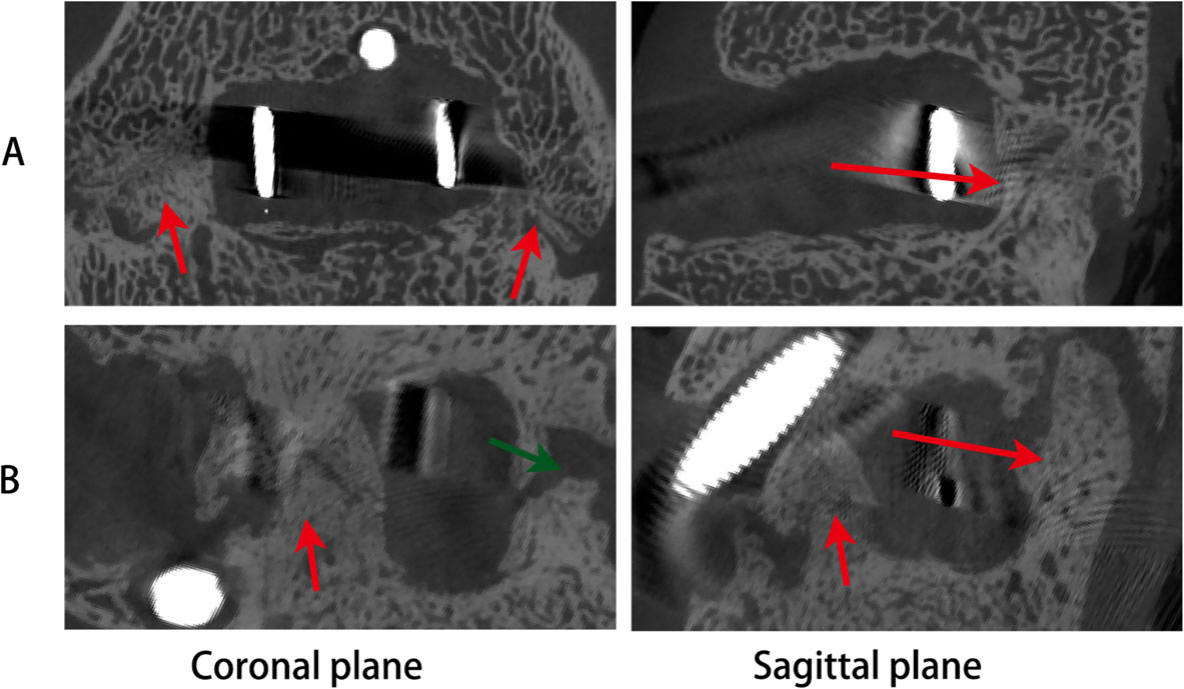
3D Micro-CT reconstruction images at 24 weeks after operation in the coronal and sagittal planes for the uncovertebral joint fusion cage (**A**) and the non-profile cage (**B**). A large amount of bone formation and definite fusion were detected in the uncovertebral joint (arrows, coronal plane in **A**) and behind the uncovertebral joint fusion cage (arrow, sagittal plane in **A**); bone formation was also detected inside and outside the non-profile cages (arrows, in **B**)

Results of the micro-CT scan and 3D reconstruction images (Fig. 7) were consistent with previous CT scans. In group A, 3D reconstruction images in the coronal planes of the cages show obvious bone formation in the region of the bilateral uncovertebral joint and images in the midsagittal planes of the cages demonstrate different amounts of bone formation in front and behind of the cages in the intervertebral space. In Group B, 3D reconstruction images in the coronal planes and in the midsagittal planes of the cages show different amount of bone formation inside and outside the cages (front, back and localized in the region of the bilateral uncovertebral joint). The bone volume fraction (BV/TV) in Group A was significantly higher than Group B (Group A: 23.59 ± 4.43%; Group B: 16.16 ± 4.21%, *P* < 0.05).

### Histological analysis

**Fig. 8:**
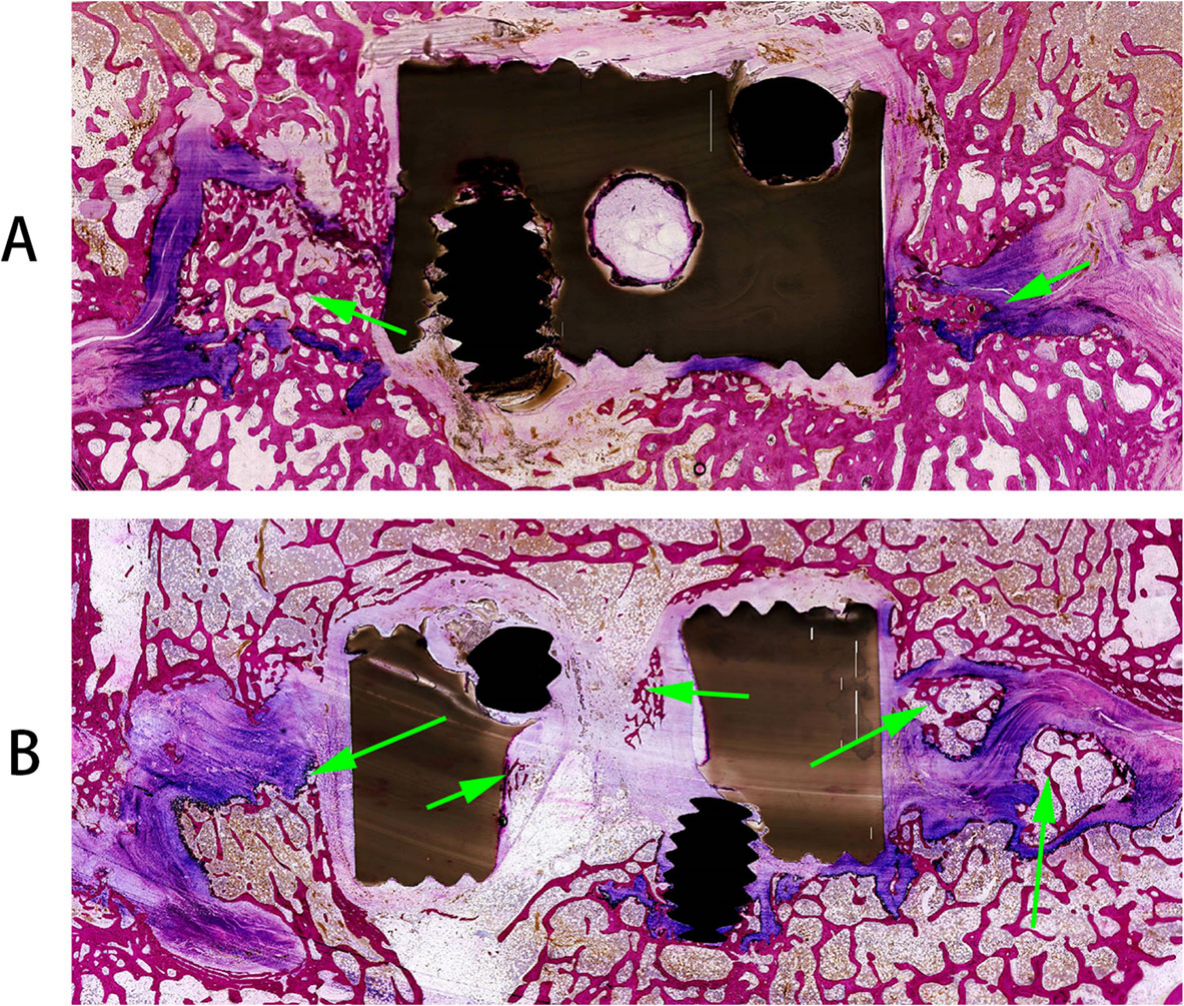
Undecalcified histological images at 24 weeks after operation in the coronal plane section using Stevenels blue and Van Gieson’s picro fuchsin staining for the uncovertebral joint fusion cage (**A**) and the non-profile cage (**B**). New bone formation was detected in the uncovertebral joint in Group A (arrows, in **A**) and localized bone formation was also detected in the uncovertebral joint in Group B (long arrows, in **B**) even without bone grafting in this region. A small amount of new bone formation inside the non-profile cage was observed (short arrows, in B)

Histological analysis was consistent with the results of micro-CT scans. Histological sections in the coronal plane exhibit obvious new bone formation in the region of bilateral uncovertebral joints with good continuity in Group A (Fig. 8A). Various amounts of bone formation inside the cages could be observed in Group B, through histological section in the coronal plane. Localized bone formation was observed in the region of the bilateral uncovertebral joint in Group B while the amount of bone formation was not obvious compared with Group A (Fig. 8B). After exclusion of the PEEK and screw, quantitative analysis indicated a mean of (32.37 ± 8.89) % trabecular bone area fraction in Group A, compared with a mean of (25.12 ± 8.32) % in Group B (*P* < 0.05).

## Discussion

ACDF has become one standard of care for treating cervical disc disease in symptomatic patients and the endeavors to refine the procedure have never stopped since its first description. In the present study, we designed a novel fusion cage with bone grafting in bilateral uncovertebral joint and conducted a preliminary test for its safety and efficacy. The evaluation of a new cervical interbody fusion cage necessitates an appropriate animal model for preliminary testing. The ideal animal model for spinal fusion should be similar to human cervical spine anatomically and be able to recapitulate biomechanical properties, be genetically homologous, reproducible and easy to raise. Although several animals such as rats, rabbits, cats, dogs, goats, sheep, swine, cattle and primates, have been reported as animal models for spinal fusion, there is no ideal cervical fusion animal model available [16]. Goats are suitable and frequently used models for cervical fusion because their postures and kinematics of the head and neck are similar to humans. Goats also share common anatomical features and biomechanical properties with humans, are easy to purchase and have low housing costs [5–7, 17, 18]. In this study, a well-established goat cervical fusion model was used to explore the safety and efficacy of the uncovertebral joint fusion cage.

Even in the absence of ideal criteria at present, the combination of X-rays with flexion-extension X-rays and CT scans was frequently used for determining clinical fusion quality after a cervical spine surgery [19, 20]. For animal studies, several methods with different advantages and limitations are available for evaluation of spinal fusion including X-rays, CT scans, magnetic resonance imaging (MRI), micro-CT scans, biomechanical tests, histologic analysis, bone mineral density tests and other uncommon methods. In the present study, X-rays, CT scans, micro-CT scans and histological analysis were selected for determining spinal fusion quality. The critical time points for this study were 12 and 24 weeks for evaluation of fusion [21–23].

Results from CT scans, micro-CT scans and histological analysis in this study consistently demonstrate that the uncovertebral joint fusion cage was effective for achieving early bone formation and the uncovertebral joint fusion in the goat model. Even without grafting in the region of the uncovertebral joint in Group B, spontaneous new bone formation in the uncovertebral joint region was confirmed by micro-CT scans and histological analysis. Similarly, bone formation and bone bridging were also observed in front and back of the cages in some goats from both groups. New bone formation in the region without bone grafting in the operation may be attributed to osteogenesis induced by spontaneous hematoma [24]. No screw loosening, screw breakage, cage displacement or subsidence could be observed for the uncovertebral joint fusion cage. These observations indicate that PEEK cages combined with direct titanium alloy screw fixation is safe for this new cage. Previous biomechanical test in goat cervical specimens suggested that the uncovertebral joint fusion cage can provide comparable initial extension/flexion stability and better lateral bending and axial rotation stability compared with the non-profile cage [25]. The wings of the uncovertebral joint fusion cage were designed to prevent the grafted bone from sliding backwards into the spinal canal or intervertebral foramen. In fact, no fragments of grafted bone slid backwards into the spinal canal or the intervertebral foramen were observed in this study. The procedure of the uncovertebral joint decompression and grafting should be carefully investigated in future studies. The vertebral artery in the transverse foramen and the spinal nerve root in the intervertebral foramen were close to the uncovertebral joint, and the operative procedures in this region might cause injury to the vertebral artery and the nerve root [26, 27]. In this study, no vertebral artery injury occurred during the surgery and the no obvious nerve injury was observed in these goats implanted with the uncovertebral joint fusion cages.

The fusion rates in control group of our study were 41.7% (5/12) and 58.3% (7/12) at 12 and 24 weeks, respectively. In other studies, which used the same criteria for the evaluation of interbody fusion based on CT scoring in goat model, autologous iliac bone with titanium plate and screws fixation was performed as the control group and the fusion scores both in 12 and 24 weeks were higher than the present study [28, 29]. Another goat study used PEEK cage filled with an autologous graft in the control group and 2/6 indicated arthrodesis with solid bone bridging the fusion area through radiographic analysis after 12 weeks [7]. Due to the differences in the surgical segment, interbody treatment, evaluation tool and endpoint, it is difficult to directly compare the fusion rate with other studies. However, a literature review of sheep fusion outcomes for control groups showed that the results of fusion in ACDF were similar regardless of interbody treatment or assessment method [23]. The fusion scores consistently indicated little to moderate bone formation with a non-bridging bony response from a single endplate at 12 weeks postoperatively.

Spinal fusion was defined as union of two or more vertebrae, and was firstly reported by Fred H. Albee [30] for the treatment of Pott’s disease. Even without a clear mechanism underpinning the uncovertebral joint fusion at present, the possible reasons of early bone formation in uncovertebral joint may be as follows: First, the shorter length. Previous anatomical studies have shown that the distance of the uncovertebral joint is about 2 mm, which is much shorter than the central height (about 5 to 8 mm) of the intervertebral space in the cervical spine [31]. This may have similar mechanism with fracture healing, which is influenced by the gap size [32]. Second, special biomechanical environment. Micro-motion existed after ACDF, and stress shielding of the central region inside the traditional cage was more obvious than the uncovertebral joint region. During cervical degeneration, the stress of the uncovertebral joint increases continuously and osteophytes of uncovertebral joint are common when the intervertebral disc degenerates and the height of the intervertebral space is lost [33]. In addition, paravertebral ossification is prone to happen at uncovertebral joint because of the mechanical stimulation during cervical movement [11]. These phenomena probably suggested that the uncovertebral joint area have a subtle biomechanical environment for bone formation. Third, better blood supply and easier haematoma formation. Uncovertebral joint is close to the vertebral artery, muscles and other soft tissues. The blood supply of the uncovertebral joint may theoretically be better than the endplates and should be verified in future vascular anatomical studies. Thus, it is likely that bilateral cavity in the uncovertebral joint without impediment from the cage body is easier for haematoma formation than the central cavity inside the traditional cage. Haematoma formation is important for fracture healing as it contains cells with osteogenic potential in local hematomas that can also induce the migration and differentiation of peripheral stem cells to the fracture site. These stem cells can differentiate into fibroblasts, chondrocytes, and osteoblasts to participate in callus formation [24, 34, 35]. The fibrous network structure formed after organization of the hematoma can allow cells and capillaries to grow and play a role in bone conduction. The invasion of capillaries and microvessels provides nutrition for osteoblast precursor cells, osteoblasts and chondrocytes, which guarantees bone reconstruction and fracture healing [34, 36–39]. These theoretical inferences and assumptions should be carefully verified by future molecular, cellular, anatomical, and biomechanical studies.

However, the current study has several limitations. First, as a preliminary animal study, the number of experimental animals was relatively small and the results should be verified by future studies with large sample size. Second, biomechanical test was not selected in this study. The finite element analysis and stability in three modes of motion on human cadaveric specimens needed to be employed in future studies. Third, the critical time point for histological analysis in this study was limited to only 24 weeks after operation, which failed to conduct continuous histological observation at early time points. Fourth, blinding was not used in the radiological and histological assessments because of the different shapes of two cages. Bias may occur in the evaluation of fusion quality using interbody fusion grading system in CT scans. Nonetheless, the distinction between Grade 1 and Grade 2 was evident in CT images, namely non-fusion and fusion, and the quantitative analysis in Micro-CT reconstruction images and histological images were less affected by non-blinding. Fifth, in early ACDF procedure, trimmed autologous iliac bone is grafted in the center of intervertebral space to restore the height and increase the stability. The cervical interbody fusion cages, designed to improve initial stability and supporting strength, continue the central grafting method of early ACDF by retaining the bone grafting cavity in the center of the cage. The main purpose of bone grafting inside the cage is to improve the fusion rate rather than the stability. Thus, on the basis of good biomechanical environment provided by interbody fusion cage itself, the transformation of bone graft sites may be one of the avenues worthy of consideration to improve the fusion rate. From grafting inside the traditional cage to grafting in the uncovertebral joint, this may be a new exploration and a new step in ACDF, but this study failed to clearly demonstrated the mechanism of the uncovertebral joint fusion.

## Conclusions

Preliminary results from CT and micro-CT scans, and the histological analysis consistently indicated that uncovertebral joint fusion cage was effective for achieving early bone formation and uncovertebral joint fusion in the goat model. The uncovertebral joint fusion cage is safe with no increase in serious complications such as vertebral artery injury and nerve injury. As a new exploration in ACDF, uncovertebral joint fusion should be verified by future studies with larger sample size, and the mechanism of uncovertebral joint fusion should be more clearly elucidated by further molecular, cellular, anatomical, and biomechanical studies.

## Data Availability

The datasets used and/or analyzed during the current study are available from the corresponding author on reasonable request.

## Abbreviations

ACDF: Anterior cervical discectomy and fusion
Zero-P: Zero-profile anchored spacer
PEEK: Polyetheretherketone
VOI: Volume of interest
BV/TV: Bone volume fraction
MRI: Magnetic resonance imaging
